# Chemical Analysis of Vaccine Virus

**Published:** 1801-11

**Authors:** Tho. Creaser

**Affiliations:** Surgeon. Bath


### *To the Editors of the Medical and Phyfical Journal.*

Gentlemen,

I Have thought it worth while to fend you a tranflation of the account of a chemical analyfis of Vaccine matter, contained in the "Traité Hiftorique et Pratique de la Vaccine," by Moreau, of Paris; a work which, in my opinion, exhibits a vaft difplay of philofophical argument, and extenfive information.

It

It is eminently worthy of the attention of thofe who are interefted on this important fubject.

There is perhaps no part of the living fyftem which is more remote from illuftration by the prefent ftate of chemical phyfiology, than the compofition and action of morbid and animal poifons; but the afferted analogy between vaccine matter and that of hydatids, may lead to experiment, or at leaft to conjecture. I am, &c.

*Bath, Oct.* 14, 1801. THO. CREASER, Surgeon.

---

" WITHOUT fuppofing that the chemical analyfis can throw any light on the mode of action of Vaccine matter, Citizens Huffon and Dupreyfen have very carefully made this analyfis. Here follow the refults which they have had the goodnefs to communicate to me.

" Expofed to the air and applied on a fmooth furface, vaccine matter dries quickly without lofing its tranfparency, becomes as hard as glafs, and peels off, adhering like a varnifh to the fubftances on which it is applied; it oxydates iron. If it is left to dry on the vaccine puftule, it takes the fhape of fmall hard globules. When fluid it is eafily diffufible in water. It is capable of the fame when hardened. There are inftances that two months prefervation of dry vaccine matter has in no degree weakened its reproductive and prophylactic powers when it has afterwards been diffolved in water. Expofed to the fire, it at firft thickens, exhales a flight fmell of carbonate of ammonia, and quickly changes itfelf into a light porous coal. It does not alter the colour of fyrup of violets, or of tincture of turnfol. Treated by alkohol, nitrate of mercury, nitrate of filver, nitrous acid, it gives a white precipitate which is not diffolved by potafh, or the muriate of ammonia. Sulphuric acid concentrated, oxalic acid, the vapour of oxygenated muriatic acid, potafh, barytes, muriate of ammonia, have no kind of action on it, not altering in any manner its exterior qualities. It appeared to us that the vaccine matter had *an analogy distinctly marked* with the matter of hydatids.

" It refults from thefe experiments that it is compofed of water and albumen, of whofe proportions we are ignorant."

Moreau. Traité Hiftorique et Pratique de la Vaccine, Paris. 1801.

